# The Ethical, Care, and Client-Caregiver Relationship Impacts Resulting From Introduction of Digital Communication and Surveillance Technologies in the Home Setting: Qualitative Inductive Study

**DOI:** 10.2196/47586

**Published:** 2023-11-03

**Authors:** Hans-Peter de Ruiter, David Clisbee, Rebecca Houston, Ingela Skärsäter

**Affiliations:** 1 College of Allied Health and Nursing Minnesota State University Mankato, MN United States; 2 Department of Computer Information Science Minnesota State University Mankato, MN United States; 3 Minnesota State University Mankato, MN United States; 4 Halmstad University Halmstad Sweden

**Keywords:** home care, caregivers, ethical implications, communication technology, surveillance technology, public health nursing practices, digital vulnerability, care of the elderly

## Abstract

**Background:**

Embedding communication and surveillance technology into the home health care setting has demonstrated the capacity for increased data efficiency, assumptions of convenience, and smart solutions to pressing problems such as caregiver shortages amid a rise in the aging population. The race to develop and implement these technologies within home care and public health nursing often leaves several ethical questions needing to be answered.

**Objective:**

The aim of this study was to understand the ethical and care implications of implementing digital communication and surveillance technologies in the home setting as perceived by health caregivers practicing in the region of Halland in Sweden with clients receiving home care services.

**Methods:**

A questionnaire was completed by 1260 home health caregivers and the written responses were evaluated by qualitative inductive content analysis. The researchers reviewed data independently and consensus was used to determine themes.

**Results:**

This study identified three main themes that illustrate ethical issues and unintended effects as perceived by caregivers of introducing digital communication and surveillance technologies in the home: (1) digital dependence vulnerability, (2) moral distress, and (3) interruptions to caregiving. This study highlights the consequences of technology developers and health systems leaders unintentionally ignoring the perspectives of caregivers who practice the intuitive artistry of providing care to other humans.

**Conclusions:**

Beyond the obtrusiveness of devices and impersonal data collection designed to emphasize health care system priorities, this study discovered a multifaceted shadow side of unintended consequences that arise from misalignment between system priorities and caregiver expertise, resulting in ethical issues. To develop communication and surveillance technologies that meet the needs of all stakeholders, it is important to involve caregivers who work with clients in the development process of new health care technology to improve both the quality of life of clients and the services offered by caregivers.

## Introduction

### Background

Recent home health care technology advances produced promising results for health care systems, including improved operations efficiency and collating real-time information. Robust data sets provide the potential to treat more clients while expending less effort. This comes amid a historical moment of caregiver shortages and an aging population. In the hustle to develop technology for the home care setting, taking time to address ethical implications and the potential for unintended outcomes becomes deprioritized. The home is the center of clients, in which care providers enter as outsiders by introducing technology that can medicalize their clients’ lives [1]. In this study, we explored the ethical and care implications as perceived by caregivers as a result of implementing digital communication and surveillance technologies in the home setting. These conflicts create an unproductive disruption to the human art of caregiving, which refers to caring as a difficult pursuit characterized by the importance of relationships and experiential knowledge. Having a precise definition of the art of caregiving would be a paradox. Cathleen Jenner stated: “The art of nursing is the intentional creative use of oneself, based upon skill and expertise, to transmit emotion and meaning to another. It is a subjective process that requires interpretation, sensitivity, imagination, and active participation” [2]. This essence of the artistry of caregiving and the functionality of home health care technology are not aligned.

In recent decades, home health care settings transformed from human dwellings with little technology to data-rich environments embedded with digital tools to support efficient care delivery [3-5]. However, despite the rapid deployment of technical resources for providers, the disconnect between technology development priorities and the art of caring persists [6-8]. Many consequences result from not embedding caregivers in the development process [7-9]. In addition, distrust of technology is compounded by repeat glitches, moral distress created by unanswered ethical questions, and doubt regarding professional expertise [1,8]. Caregivers struggle to see how technology supports the human aspects of their work [8,10], which includes the degree to which the implementation of technology aligns or conflicts with their professional values [11,12].

There is a growing body of literature focused on the implementation of technology in the home care setting. The main reasons for moving care from organizations such as hospitals to the home include allowing people with chronic illnesses to gain more control of their lives [10], along with the efficiency of care such as remote problem-solving [8], cost savings, and shortfalls in health workers [13]. New technologies provide preliminary answers to home health care challenges, yet these technological “solutions” come with ethical questions. Notably, the rise of surveillance technology integration into everyday objects such as smartphones and watches has become ubiquitous. This form of surveillance is referred to as ambient intelligence and ubiquitous computing [9]. However, the ethical and unintended effects are not known.

There is a strong call to advance ethical inquiry while implementing technology [3,7,14]. This evaluation should incorporate not only the client and family perspectives [8,15,16] but also the caregivers’ perspectives [6].

The literature described and the starting point for this study illustrate how implementing technology in the home care setting has allowed for more efficiently managing care delivery from an institutional perspective [17]. However, this research specifically demonstrated the impact on caregivers and the subsequent loss of the art of care and ethical concerns [17].

Note that we have chosen to consistently use the term “client” throughout this article for readability purposes; however, the term “clients” also includes persons who could be referred to as “patients” in practice.

### Objective of the Study

The aim of this study was to understand the ethical and care implications as perceived by caregivers because of implementing digital communication and surveillance technologies in the home setting.

Research questions resulting from this aim were: (1) What are caregivers’ key ethical and care concerns regarding using digital communication and surveillance technologies in caregiving? (2) What are the emotional and psychological implications experienced by caregivers due to using digital communication and surveillance technologies? (3) How do caregivers perceive the impact of digital communication and surveillance technologies on the overall quality of care provided to their clients?

### Philosophical Framework

The philosophical foundation of this study is rooted in the works of Jacques Ellul and Sherry Turkle. Ellul [18] focused much of his work on trying to understand the impact of techniques or technology on humans, with an emphasis on the effect rather than the intent (which he considered efficiency) [18]. Sherry Turkle [19] refers to the purpose of her work as understanding what technology does *with* us rather than *for* us.

This study focuses on the ethical and unintended effects of technology used in the home setting by professional caregivers. One might also refer to this as the “shadow side” of implementing technology in a client’s home. The purpose of this perspective is not to position ourselves as Luddites that wallow in a romantic notion of years bygone; instead, our curiosity is based on a belief that this knowledge is essential as technology evolves toward ultimate usefulness. We witness this when the purpose and function of technology are in ethical alignment with the values of its users and the people it is intended to serve. A positive development is stalled in looking away from understanding the ethical and unintended consequences.

In summary, to fix something, one must first know what is broken and according to whose perspective. The understanding of the limitations of technology forms the foundation for developing improvements and solutions and results in more user-friendly technology where the purpose of that technology is clear to all who engage with it, directly or indirectly. The intent resulting from this research is to help narrow the gap between the benefits and utility technology development offer with the limitations experienced by caregivers and their clients, who are the supposed beneficiaries of the technology.

## Methods

### Design and Setting

The study is part of a larger project, Digga Halland, a European community–funded initiative focused on implementing digital technology in the home-health setting to make care delivery more efficient in the health care organizations of six municipalities and two hospitals. The Digga Halland project was initiated in 2018 in southern Sweden within a region with 336,748 participants, and data collection using surveys started in 2019.

The focus of the Digga Halland project was to address current and future challenges in the health care sector, such as the aging population and predicted scarcity of care providers [20]. Digital services and systems were considered essential to meeting these challenges and creating equal health care with high quality. This study focused on the survey data collected during the Digga Halland project, and specifically on the digital vulnerabilities of clients and caregivers as expressed by the caregivers in the survey data. For an overview of the Digga Halland Project, refer to Ruiter and Skärsäter [21].

### Procedure

Approximately 15,000 people were employed at health care organizations in the region of Halland, approximately two-thirds of whom agreed to participate in the Digga Halland Project. A web-based baseline survey was sent to 9161 people in February 2019, with a response rate of 31.43% (n=2879). Of the participants, 87.98% (n=2533) were women, 84.99% (n=2447) had Sweden as their country of birth, and 69.99% (n=2015) were >41 years of age. Moreover, among the 2879 respondents, 86.00% (n=2476) had a high school or university education and 48.00% (n=1382) worked as nursing assistants. The professional care providers in this study included nurses, physicians, occupational therapists, physical therapists, social workers, and unit managers. A follow-up survey was sent out in February 2020 to 9983 people with a response rate of 35.00% (n=3494), comprising 89.01% (n=3110) women and 71.01% (n=2481) above 41 years of age; 65.00% (n=2271) responded to baseline measurement and follow-up requests after 1 year.

### Data Collection

An overall web-based survey was developed, including 20 questions comprising six focus areas highlighted in the project: digital competence, conditions in the workplace, safety and ethical consequences, participation, horizontal criteria, and background issues. The purpose of the survey was to obtain a comprehensive understanding of the implementation of health technology in the home. However, this study’s primary focus was limited to the ethical and care consequences and understanding the impact of technology on the caregiver-client relationship. The caregiver participants were asked to give written responses to the following: What ethical or care delivery problems have you (caregivers) experienced relating to (1) the implementation of digital communication and surveillance technologies and (2) issues resulting from the everyday use of digital tools/services/aids when providing care to clients. A total of 1260 written responses were obtained, including 530 in 2019 and 730 in 2020.

### Analysis

The interview data were analyzed using qualitative inductive content analysis to examine patterns and themes to understand the meaningful content related to the aim of understanding the ethical and care implications as perceived by caregivers [22]. The analysis began with the researchers’ immersion in the transcribed data. First, the authors read all written (N=1260) transcriptions several times to recognize and highlight the central meaning of the responses. This made it possible to identify relevant sentences and phrases and divide the data into meaning units labeled with codes. The following steps were to merge the codes into subthemes, which were then grouped into three more prominent main themes. Next, the authors created a key map showing the relationships between the meaning units, themes, and subthemes. Data were independently reviewed by two researchers and consensus involving a third researcher was used to determine themes.

### Ethics Considerations

The study was conducted according to ethical standards [23] and was approved by the Swedish Ethical Review Authority (Dnr 2019-03263). The participants received written and oral information about the study and gave their written consent to participate.

## Results

### Main Themes

The results of this study are rooted in the curiosity about caregivers’ perceptions of how the newly introduced technology resulted in ethical concerns (research question 1), emotional and psychological implications on caregivers (research question 2), and impacted patient care (research question 3). This resulted in the identification of three themes that exemplify how technology impacts caregivers’ abilities to offer care that they perceive as safe and aligned with their professional values:

1. Caregivers experienced what we refer to as *digital dependence vulnerability,* which is defined as a “condition of susceptibility to harm that stems from the use of digital technologies” (page 834) [24].
2. *Moral distress* is associated with how technology influences caregiver capacity to perform previously established care routines grounded in their professional values and expertise.
3. Technology presented an *interruption in caregiving,* where there was minimal harmony in how caregivers interacted with clients while using the technology.

Each theme has subthemes that reflect different facets of ethical and other care issues perceived by caregivers when the technology was introduced into the home care setting.

### Digital Dependence Vulnerability

#### Theme Overview

The caregivers’ key ethical and care concern regarding using digital communication and surveillance technologies in caregiving (research question 1) was *digital dependence vulnerability*.

Digital vulnerability is rooted in dependence on technology. The introduction of the internet has propelled this dependency. When digital technology loses its functionality, it leads to significant vulnerability levels. Entire organizations and communities shut down, which results in massive disruptions at a societal level. This study identified a more micro level of digital vulnerability in which the expected care delivery was interrupted or made difficult. This theme focuses on how the introduction of technology has made care more vulnerable and contributed to a higher risk of harm because of the dependency on technology.

#### Risk for Victimization and Harm Toward the Client

Digital technology makes clients more susceptible to harm and risk of abuse due to the increased risks associated with having large amounts of information stored on the internet, and when accessed by cyber criminals, leads to previously unknown threats. For instance, a caregiver reported a client’s concern regarding using digital locks by home care staff, creating a potential security problem. In addition, many digital technologies leave footprints that malicious actors exploit: “the digital locks allow neighbors or even thieves to see which people in the area have home care easily and perhaps use that information” [Participant 39].

From an institutional perspective, efficiency, increased productivity, and risk management are often priorities. Technological systems such as electronic locks are developed to promote these priorities. However, when these systems are introduced, the predominant focus is their effectiveness, while the client’s concerns about being vulnerable because of the loss of control and who can enter their home are real.

#### Caregiver Concerns Regarding Consent

Using new technology (eg, SMS text messages) to communicate with health personnel complicates determining if appropriate client consent was obtained and if close family relatives were given permission to disclose confidential information on behalf of their loved ones. Along the same line of consent issues is another standard technology, group distribution lists, which have been found to make it easier for confidential information to be breached. Clients should be made aware of everyone they consented to receive information. With readily available communication methods, many unknown people have entered the “client room.”

You have to think about what is written in, eg, SMS that is sent out to everyone in the staff group, eg, change of port code number, not appropriate for everyone to take part of such info or, eg, SMS about deaths names that have gone out to everyone in the staff group.

Participant 35

The addition of communication technology to the home setting has led to many invisible actors being present. Messages regarding clients are accessible to several people, many of whom neither the caregiver nor the client know. Caregivers who value client autonomy and respect their right to consent to share information experience stress when they do not know who may have access to client information. This is risky, for example, for people with hidden identities.

We need customer telephone numbers, but the question is where they can be stored when we use digital services where we do not have complete control over personal data.

Participant 6

I see a major problem because there needs to be a routine for how confidential individuals should be treated in all systems.

Participant 44

The ease of accessibility and data transfer has also increased the risk of breaching confidentiality. Many more actors within institutions receive access to client data to do their work (eg, risk management, billing, and management). Limiting access to data is difficult since the same data can be used for multiple purposes. For example, a client’s phone number could be essential for a direct caregiver; however, it would surpass the need to know for a person doing chart audits as part of a quality improvement plan. Another issue is that the direct caregivers, who traditionally were the holders of the medical chart, need to know who is accessing data, thus resulting in a perception that the client’s confidentiality is at risk and concerns that information is accessed without the client’s or family’s consent.

#### Technology and Change Agent in Power Relations

Technology has entered the space between caregivers and clients to the degree that it impacts conversations and relationships. For example, technology such as electronic records turns the caregiver into an interface between the client, who has become a “data point,” and the institution interested in harvesting all client data, who has become the “data set” for institutional purposes. One of these purposes, to offer quality care and lead to satisfied health care users, is in line with the caregivers’ goals. However, multiple other goals such as billing, risk management, and institutional safety align less with the primary purposes of the caregiver. These changes have led to a shift in power relations.

That digital replaces a person’s conversation, a person’s meeting, that many of the clients I meet do not belong to the generation that knows of, or the strength to absorb information about the digital. That many of the clients I meet are cognitively impaired and do not understand what happens when it happens digitally.

Participant 97

Caregivers need transparency about technology in the home to understand its impact on the client. The technology creates a sense of data collection through questioning, leading to a lack of understanding and affecting the client-caregiver relationship. The data points may meet institutional priorities, but the client lacks the experience of being cared for. Additionally, clients wonder why data were being collected, what they would be used for, and who can access them. What used to be perceived as a conversation with one’s caregiver has become more of an interrogation through a set of required questions.

The digital dependence vulnerability was perceived by caregivers in this study as putting clients at increased risk due to the “visibility” of their vulnerability; increased exposure of their confidential information to many more known and unknown parties; and a change in the power relationships between the institution, client, and caregiver.

### Moral Distress

#### Theme Overview

Moral distress occurs when people perceive an imbalance between their values and what they are expected to do, such as the roles and responsibilities of caregivers regarding how they use new technology. Caregivers deal with conflicting values, perceiving their actions as conflicting with what they consider best practice. This finding is an answer to the second research question, which explored the emotional and psychological implications experienced by caregivers. Three subthemes that emerged illustrate the moral distress that resulted in the caregivers.

#### Balancing Between Institutional and Client Needs

Technology provides new, innovative improvements to medicine. However, there are numerous downsides to the amount of time technology consumes. The utilization and management of technology require additional time, which is often taken away from the attention given to clients. This time spent on technology can be experienced as the “client’s time” taken away from direct client care, interpreted as inattention to the client or misunderstood when the caregiver’s focus is on the technology. Caregivers receive adverse reactions from clients due to these misunderstandings of bedside technology use, which strains the trust in this fragile relationship*:* “What other colleagues and I have reacted to is that digital work ‘steals’ more and more time from the client’s granted hours. It is not the case that someone has more time than is needed” [Participant 123].

Care is shifting from direct client contact to technology-mediated care, with the demand for technology increasing caregivers’ stress. Caregivers were deeply aware that their use of technology was affecting their ability to interact with clients fully.

#### Caregiver Moral Distress

From the lens of the caregiver, there is a different level of moral distress they experience when caring for others. Determining the boundaries between the caregiver’s mission to support clients and comply with institutional requirements is ongoing, which increases the risk of harm to clients if data entered by the caregiver are used for other purposes such as insurance coverage or paid caregiver hours. In addition, navigating an increasing rate of change in their professional environment impacts the feelings of competency that caregivers experience in their level of competency. Nurses revert to Patricia Benner’s [25] levels of expertise (novice to expert) and find themselves reapproaching the novice level because of their self-doubt in their technology-mediated caregiving*:* “...you experience that the training for new things is too fast. If you were not good before, then you feel entirely gone. One can only hope that the colleagues understand and take the time to help” [Participant 59].

Caregivers feel they have lost control of their ability to determine how to practice when doing their work. As a result, they cannot act in the way they believe is right or, at times, think that institutional directions squash the actions they ought to do, such as when or when not to give a medication to a client.

#### Surveillance Caregiver Issues

Technology allows constant work monitoring. Perpetual oversight gives the institution more control over caregiver practices; however, this also comes with a shadow effect. Communication technology not only serves to monitor clients but also caregiver actions. A work environment with endless surveillance leads to caregiver stress. Caregivers experience reduced freedom and decreased control over their own work.

Now, it does not work because if you sign [medication list] outside the time frame, yes, then there will be deviation reporting, which is linked to the threat of losing your delegation if you get too many deviations due to late signing, a problem that has arisen due to the new digital aid.

Participant 94

A work environment where all work is monitored leads to high stress levels for both the client and caregiver. Although the institutional intention is to increase productivity, quality, safety, and reimbursement, it also dehumanizes the interactions, leading to a bifurcation of consciousness in which the caregiver and client are simultaneously in two realities.

Digital aids for supervision can be good, and we often emphasize that it is good that customers are not disturbed during the night, for example. However, many people are alone, and the home care service is the only visit you get for a whole day. Is it right for that person to talk on a screen, or does it require a human visit?

Participant 112

On the one side, the reality of being able to observe real people in real time and space can be beneficial, whereas on the other side, the reality of being watched and needing to meet all the institutional requirements in a way that might not be aligned with what they are experiencing can be stressful.

### Interruption to Care

#### Theme Overview

Insights into the third research question that focus on how caregivers perceive the impact of digital communication and surveillance technologies on the overall quality of care mainly highlight the *impact resulting from the interruption to care*. The technology deployed within our study’s care environment was reported as interruptive to previously established care delivery approaches. Interruption to care can be defined as when technology negatively impacts the client-caregiver relationship or impedes what caregivers perceive as ideal care delivery. Care interruptions are barriers that have multiple effects, which include client dissatisfaction or omission or delay of care. Such interruption also results in disruptions to day-to-day workflows. Interruption to care was reflected in three subthemes: (1) functionality and usability concerns, (2) unintended trepidation, and (3) impact on care. The subthemes illustrate the breadth and complexity of technology implementation choices impacting various parts of the care continuum.

#### Functionality and Usability Concerns

The technology malfunctioning was noted as a pervasive finding. Caregivers are increasingly dependent on the reliability of technology while they provide care. When technology fails, there can be a paralysis of the workflow, completing tasks, and finding the way to the client. Caregivers perceived this technology as a cumbersome “blackbox,” meaning little was known about the technology’s inner workings and access to technical support was limited. Despite the uncertainty of how or if the technology would work the way it needed to, the requirement to use it was apparent. One concern expressed about the technology’s unreliability was expressed: “When we provide medicine with alpha e-drugs, the phones do not update, so it does not appear that the medicine is signed. There is, therefore, a significant risk that medicines will be given twice as much” [Participant 144].

Technical problems contributed to caregivers having more questions about the technology’s implementation, purpose, and effects on their abilities to perform day-to-day care duties. One respondent stated*:* “We introduce new systems but forget to implement them. In addition, they are often updated so that you do know how to use them and have to think about how to continue to use it. We have too many passwords in too many different systems” [Participant 56].

Questions created by everyday functionality problems and the overall implementation of the technology appeared to compound into additional concerns described by the following provider: “What to do if it suddenly stops working during the day? All planning is in the mobile [device], which clients to walk/cycle/drive to. Travel and work will be delayed to clients until you get in paper format where to go.” [Participant 60]

Implementing the technology was often not sought by caregivers. Instead, it was imposed without consideration of individualized and client-centered care practice.

#### Unintended Trepidation

The technology’s unreliability and unclear ethical implications created unintended yet distracting trepidation. Caregivers reported concern about information being collected. They were uncertain of its purposes or the degree to which their clients’ priorities were considered. Additionally, the caregivers felt their priorities and expertise were not considered in developing the technology.

We see all clients admitted to hospitals in the region in Lifecare [a data system]. Also, friends, neighbors, and coworkers. Everyone is required to go in and watch daily, so everyone sees everything. Extremely unethical and not confidential.

Participant 25

Have clients who live in digital exclusion. Clients who need more money to buy a smartphone, iPad, data, internet, etc. Have older clients who need more interest/ability to learn. That is a dilemma. Challenging to use digital services when clients do not have a bank ID etc.

Participant 115

That we handle our digital tasks sometimes feels more important than the well-being of the residents themselves, as digitization is seen in a unique way upwards, than what the most important work in my opinion does, what we do here and now within their homes and their well-being and values.

Participant 178

The apparent disconnect between institutional priorities represented by technology and caregivers’ concerns about the lack of value placed on their professional expertise negatively affected the quality of care as perceived by caregivers.

#### Impact on Care

Survey respondents reported that the use of digital tools contributed to (1) a barrier between the client and caregiver, (2) caregivers feeling insecure in their expertise, and (3) a disruption to the caregiver’s capacity to build relationships with clients. Digital information and communication technologies appeared to interfere with direct contact. Instead of a bidirectional client-caregiver relationship, the relationship was perceived to change to client-technology-caregiver. Further, caregivers felt a loss of what they perceive as essential caregiving, or the art of caring, based on interpersonal communication, rapport-building, and presence.

Direct contact and attention were diminished since the technology was perceived as between the client and the caregiver. This perception changes how the client is known; it is as if technology plays a primary role in determining care priorities, not the caregivers. Given this, caregivers are challenged by navigating competing priorities simultaneously, including caring for clients, using digital tools, and explaining the digital tools’ uses and purposes to clients*:* “[I am not able] to clearly understand the situation around a client without printing out the client profile. The text [in the profile] may feel impoverished (lacking detail), and misinterpretation of the situation is likely” [Participant 47].

Due to the central place technology has taken in the care delivery process, the past expertise of caregivers is often no longer needed as the technology automatically leads the caregivers through the care process. Experienced caregivers struggle with mastering new technologies. This combination of following the technologies’ “thought process” and managing the nuances of using new technologies leads experienced caregivers to feel like they perform at a novice level, whereas they previously perceived themselves as experts. Rather than expertly guiding a conversation with a client, technology guides the conversation in an impersonal order*:* “As a result of implementing technology, the opportunity for dialogue and follow-up questions is rare” [Participant 53].

Situations and relationships that were once familiar are no longer perceived in that same light. There is also fear that technology might eliminate caregivers from the home setting. Caregivers see technology’s impact on clients but cannot change this.

Personal care is suffering in an increasingly digital society, where our old people have not had time to understand the benefits of it. That everything should go faster and faster due to a lack of staff and be replaced by digitized aids and lose nursing along the way.

Participant 95

If you rely too much on digital, there is a risk that you will stop thinking for yourself. If the system does not work and all the information is there, it may not be possible to work.

Participant 184

Technology has shifted from a tool to help support care delivery to a device that drives how care should be delivered. The institution can now direct what is being done at the bedside by requiring the caregivers to document certain items. This has shifted the focus of control away from the individual care provider to the institution, resulting in caregivers losing individual control of their practice.

Caregivers experienced a loss of autonomy in care, which they consider a loss in quality in providing care tailored to the individual client. The steering of care by technology and the algorithms that fuel them only sometimes align with what the caregivers consider a priority. The shift of decision-making from the care provider to the institution, as represented in the technology, resulted in a perceived deprofessionalization, where having a unique skill set and ability to make judgments regarding how to best help clients were replaced by the requirement to respond to what is asked for by the technological devices.

## Discussion

### Principal Findings

Digital and surveillance technologies are being implemented in home care settings, with caregivers in this study experiencing the unintended consequences of those technologies in the three main areas this study focused on: (1) ethical concerns in caregiving, (2) emotional and psychological impact on caregivers, and (3) impact on caregiving. The ethical and care implications include a shift in the caregivers’ autonomy in the institution. Caregivers perceived a loss of ownership over who has access to what they communicate and how the information is shared with others. The control over what and how data are shared has shifted from the care providers to the institution. The same is true regarding the power of who controls essential information. This has moved away from the caregiver, who is a real person, to what they perceive as a faceless institution [17]. Consequently, caregivers transitioned from real people to a human interface between the client and the institution. The introduction of documentation technology has reduced autonomy in caregiver practice as this has shifted to the responsibility of institutional information and technology departments. This resulted in the ultimate control over client information being an institutional responsibility. The individual or entity responsible for overseeing the documentation requirements and regulating access to data holds the power to determine the actions that can be taken [26]. This shift of responsibility from the caregiver to the institution impacted what is considered the “art of caring,” as the caregivers are now directed by technology on how to practice rather than having documentation as a reflection of their practice.

The qualitative data obtained for this study illustrate how caregivers’ ability for relationship building can be impacted and thwarted by implementing new communication technologies. Once intimate face-to-face encounters between client and caregiver—discussing instances of nighttime restlessness, lapses in memory, and risky behaviors—has now evolved into caregivers completing impersonal tasks of logging client answers to standardized questions on standardized checklists. In several cases, neither the client nor the caregiver recognizes their communication as a meaningful conversation about health between two people due to the technology’s obtrusiveness.

The changes in caregivers’ roles imposed by technology have led to three main effects on care delivery, which are summarized in Textbox 1.

**Primary effects of technology on home care delivery.**

1. Technology has replaced the “holder of information” with the electronic health record, where all data are held. A significant change introduced in the electronic health record is that information is not stored in a stagnant place as is the case for traditional paper records. Instead, the electronic health record is set up as a spreadsheet from which data can be pulled.
2. The electronic health record can be accessed and managed by multiple actors simultaneously. This means that many invisible institutional actors have joined the bedside. Caregivers no longer know who and for what purpose their document action is being used. In addition, the fact that communication technology is housed within cyberspace, and many people need access to the record, has increased the risk of unauthorized people having access to that information.
3. The content in the documentation is changing in real time, resulting in the need for ongoing use of the record each time information is needed rather than relying on memory or paper if the technology is unavailable. This results in an inability to provide safe and accurate care for the caregivers should there be any technology disruptions.

Digital communication and surveillance technologies have brought to light ethical principles such as beneficence, nonmaleficence, and autonomy [27]. Although caregivers have an ethical and legal obligation to care for their clients, the transparency around how clinical decisions are made is diminished. Clients lose their autonomy to decide what or who has access to their information and how it is used, even though codes of ethics require caregivers to protect the safety and well-being of their clients, especially when it comes to privacy and protecting sensitive information. Caregivers could enter information, yet they cannot protect that information from being misused as it is housed and managed by the institution.

All information entered in the system can now be surveilled. This also includes sensitive information such as tax ID numbers, addresses, and information to enter clients’ homes. However, it can also include information that could impact the ability to gain life insurance, obtain employment, and other purposes. Health records can often be subpoenaed for various purposes. Understandably, the trust relationship, which is essential for diligent care, is impacted if information clients believe they give in confidence becomes a public good available to multiple institutional players and is used for purposes other than what was intended.

Due to the ability of the institution to exercise total surveillance on the work of the caregivers by being able to not only access all their information but also to automize the surveillance process by having the systems create ongoing reports to supervisors, caregivers feel increasingly morally burdened. Suppose they do not meet the institutional requirements. In that case, the threat of repercussions can conflict with the caregiver’s obligation toward the client by shifting the caregiver’s time and attention away from the client to managing the electronic communication systems. Codes of ethics speak little about the obligation toward the health organization but rather focus on the obligation toward clients; however, there is a bifurcation in what caregivers believe are their obligations and what is being asked of them. Intuitive knowledge is crucial to the art of caregiving; however, this professional knowledge becomes less valued. It is challenging when caregivers are expected to respect the principle of beneficence to the client yet receive the implied message that beneficence toward the institution is paramount.

The disruption in care resulting from implementing technology, specifically electronic communication technology, has challenged the above-mentioned ethical principles and impacted the caregiver’s ethical obligation toward beneficence, nonmaleficence, and autonomy. In addition, caregivers can no longer promise confidentiality when data are entered into the system as they have no information regarding who has access to the data and how they may be used.

### Limitations

The limitations of this study are that it was performed in Halland, a region in Sweden. In addition, the findings were based on an electronic survey sent to the caregivers. This study also might reflect the biases of the participants. The clients had no direct input into the data of this study. Thus, even though many caregivers and clients might relate to the findings, they cannot be generalized.

### Recommendations

The main recommendations (Textbox 2) based on the findings of this study focus on taking a proactive approach in not only identifying ethical issues after the implementation of technology but also including ethics evaluation as an essential element during the development phase of new technologies. Being curious about the possible ethical and unexpected effects of a new technology is critical to developing the best possible new products.

**Main recommendations for the development of technology in home care based on the study results.**

1. When developing a new technology, the focus should not be limited to the intent but also on the effect experienced by the users, in this case the caregivers. Caregivers’ knowledge of the care process and the client can offer insights into the predicted effects of the new technologies. This could be achieved through focus groups or observation of the work in real time and space.
2. When developing new technology, the priorities of all key stakeholders should be integrated. In the development, the priorities of the (1) institution, (2) client, (3) care provider, and (4) developer of the technology should be valued
  equally
  and not primarily on cost-savings aspects. As the institutions are typically in charge of developing and implementing new systems, which often include technology, this can easily result in the institution having the most input in what is created. This can result in the effects observed in this research. Using a collaborative approach can decrease unintended effects that result from doing so and the costly changes that need to be made resulting from those unwanted effects.
3. In addition to their technical education, developers of new technologies should have training in ethics and the values of the professions for which they build technology. Developing new technologies while evaluating them ethically can help avoid unwanted consequences.
4. The concept of the Art of Caring can offer a helpful framework for technology developers to understand what is important while taking care of real people in real time and space. Familiarizing health technology developers with the concepts of the “Art of Nursing” and “Client-centered care” can provide important insights into how to develop caregiver and client products from the onset.

### Conclusion

Implementing technology in the home care setting allows for more efficiently managing care delivery from an institutional perspective. As a result of the increased use of digital communication and surveillance technologies in home care and the use of electronic records, there has been a shift in decision-making away from the care provider to the institution. Clients and caregivers have been exposed to digital dependence, vulnerability, and moral distress and are experiencing interruptions to care. This has contributed to (1) a barrier between the client and caregiver, (2) caregivers feeling insecure in their own expertise, and (3) a disruption to the caregiver’s capacity to build relationships with clients. It also has resulted in a perceived deprofessionalization and the loss of the art of caring. Utilizing a unique skill set and making judgments regarding how to provide individualized care are replaced by the requirement to respond to what is asked by technological devices. From an ethical perspective, conflicts of beneficence, nonmaleficence, and autonomy have resulted.

These findings are intended to offer insights into how technology development and implementation can be more client-centered and caregiver-friendly. The benefits of technology are crucial in the advancement of care delivery. By integrating these findings and recommendations into future communication and surveillance technologies used in home settings, the increased satisfaction of caregivers and clients can be included as a benefit of technology.

## Data Availability

Public data availability is restricted due to privacy protection. However, access to the data can be provided upon request to the corresponding author.
